# Supplementary material for: Magnetic Silica-Coated Iron Oxide Nanochains as Photothermal Agents, Disrupting the Extracellular Matrix, and Eradicating Cancer Cells
Source: Cancers (Basel). 2019 Dec 17;11(12):2040. doi: 10.3390/cancers11122040 (PMC6966508; doi:10.3390/cancers11122040)
Supplement: Supplementary file 1 [file cancers-11-02040-s001.zip › cancers-641801-Supplementary.docx]

Supplementary Materials: Magnetic Silica Coated Iron Oxide Nanochains as Photothermal Agents, Disrupting the Extracellular Matrix and Eradicating Cancer Cells

Jelena Kolosnjaj-Tabi, Slavko Kralj, Elena Griseti, Sebastjan Nemec, Claire Wilhelm, Anouchka Plan Sangnier, Elisabeth Bellard, Isabelle Fourquaux, Muriel Golzio and Marie-Pierre Rols


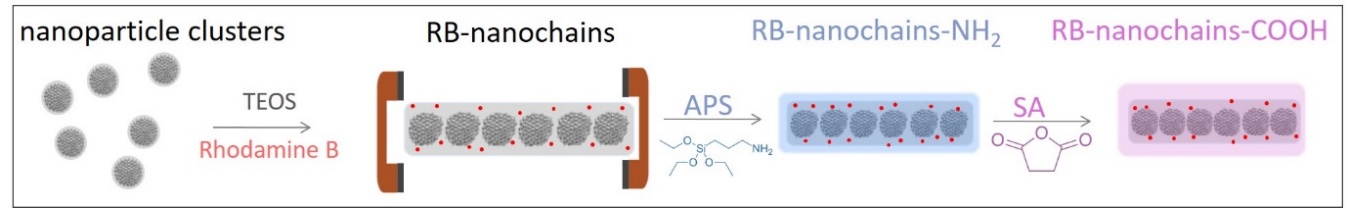

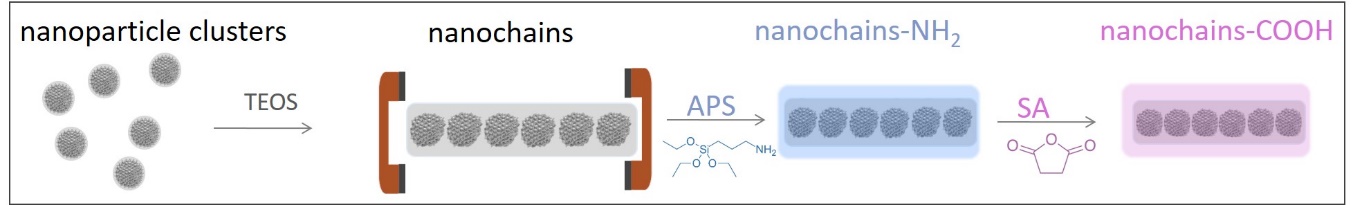


**Scheme S1.** Schematic representation of the main synthesis steps.


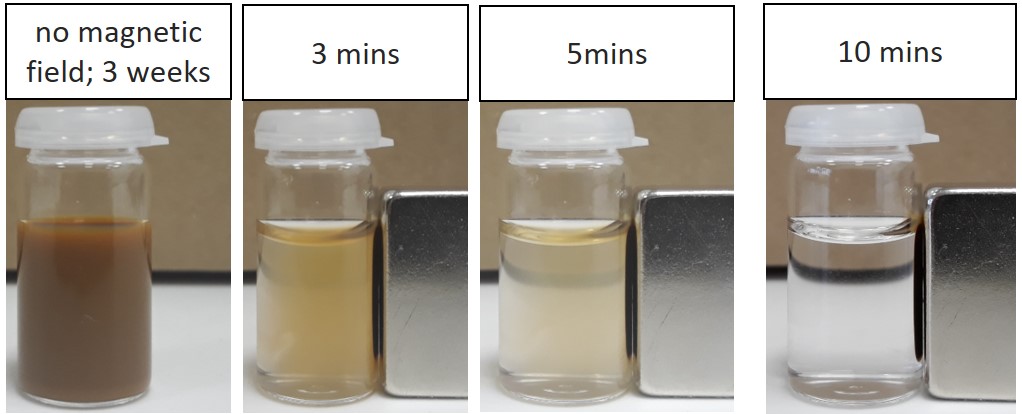


**Figure S1.** Demonstration of magnetic responsiveness of the RB-nanochains-COOH. If the suspension of the magnetic nanochains is not exposed to an external magnetic field, the nanochains remain dispersed homogeneously in the liquid for at least three weeks. Next, the photographs demonstrate rapid magnetic separation of the nanochains over time, confirming the potential applicability where magnetic guidance is desired.


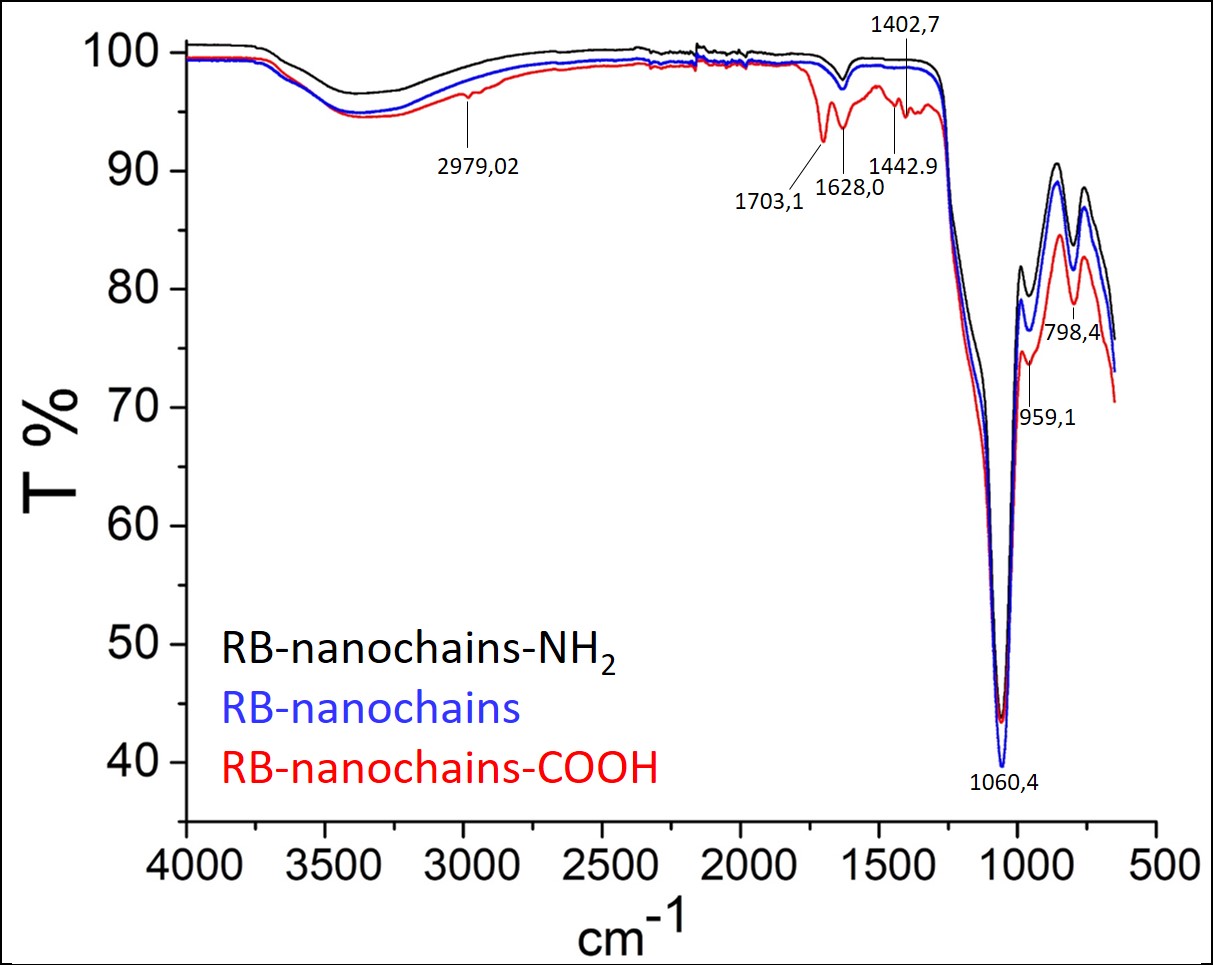


**Figure S2.** The FTIR-ATR spectra of the RB-nanochains, RB-nanochains-NH_2_, and RB-nanochains-COOH. The spectrum of the RB-nanochains-COOH shows the distinctive bands at wavenumbers 1703.1 cm^-1^, 1442.9 cm^-1^ and 1402.7 cm^-1^ confirming the presence of carboxyl group and amide bond. However, the primary amines of the RB-nanochains-NH_2_ are not well visible because they are overlapped with intensive silanol OH of silica at wavenumbers above 3000 cm^-1^.


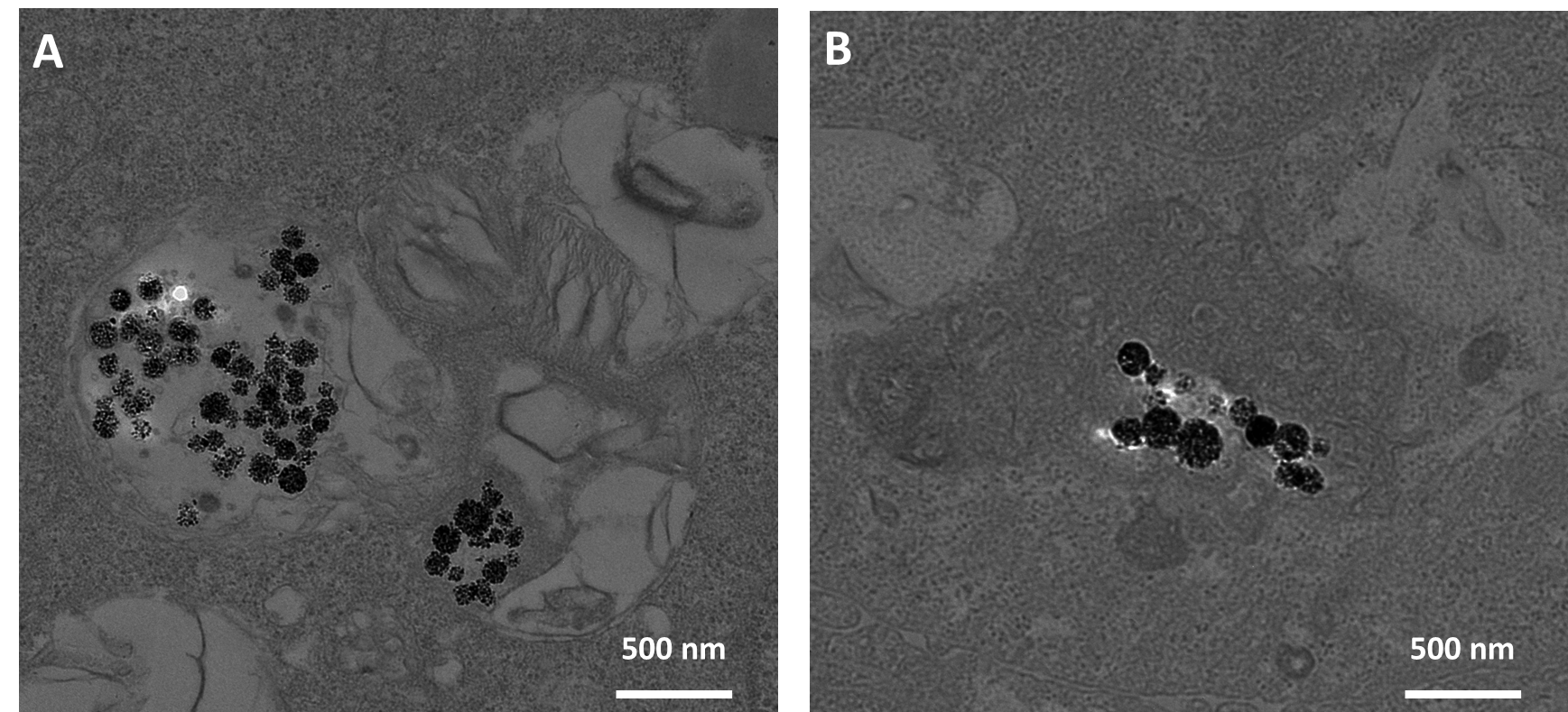


**Figure S3.** High-magnification TEM micrographs of RB-nanochains-COOH found within cancer cells: (**A**) HCT-116-wt and (**B**) HeLa rab 7 gfp.
