# Supplementary material for: Magnetic Silica-Coated Iron Oxide Nanochains as Photothermal Agents, Disrupting the Extracellular Matrix, and Eradicating Cancer Cells
Source: Cancers (Basel). 2019 Dec 17;11(12):2040. doi: 10.3390/cancers11122040 (PMC6966508; doi:10.3390/cancers11122040)
Supplement: Supplementary file 1 [file cancers-11-02040-s001.zip › Videos Supplementary.pptx]

## Slide 1
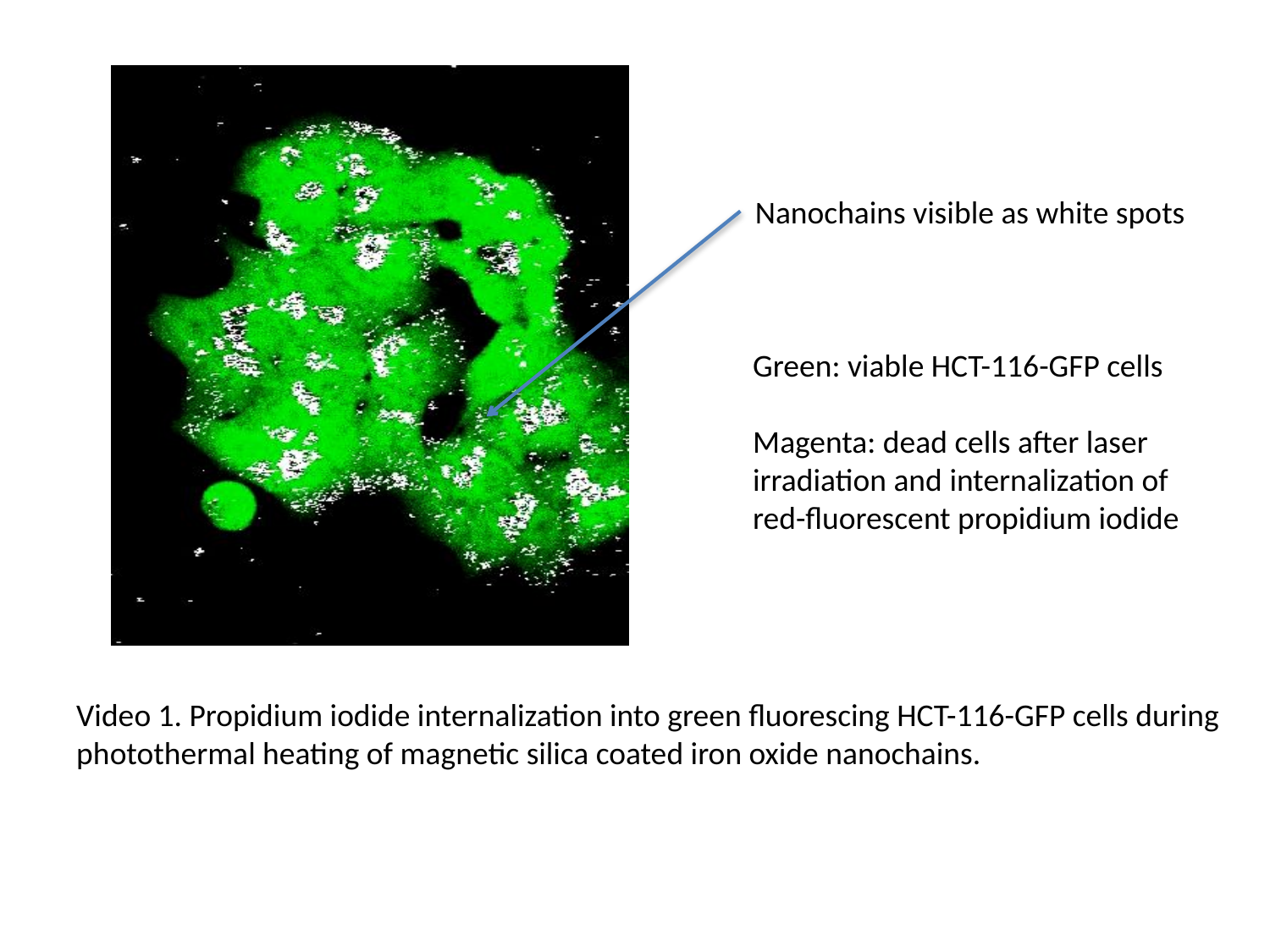

Nanochains visible as white spots
Green: viable HCT-116-GFP cells
Magenta: dead cells after laser irradiation and internalization of red-fluorescent propidium iodide
Video 1. Propidium iodide internalization into green fluorescing HCT-116-GFP cells during photothermal heating of magnetic silica coated iron oxide nanochains.

## Slide 2
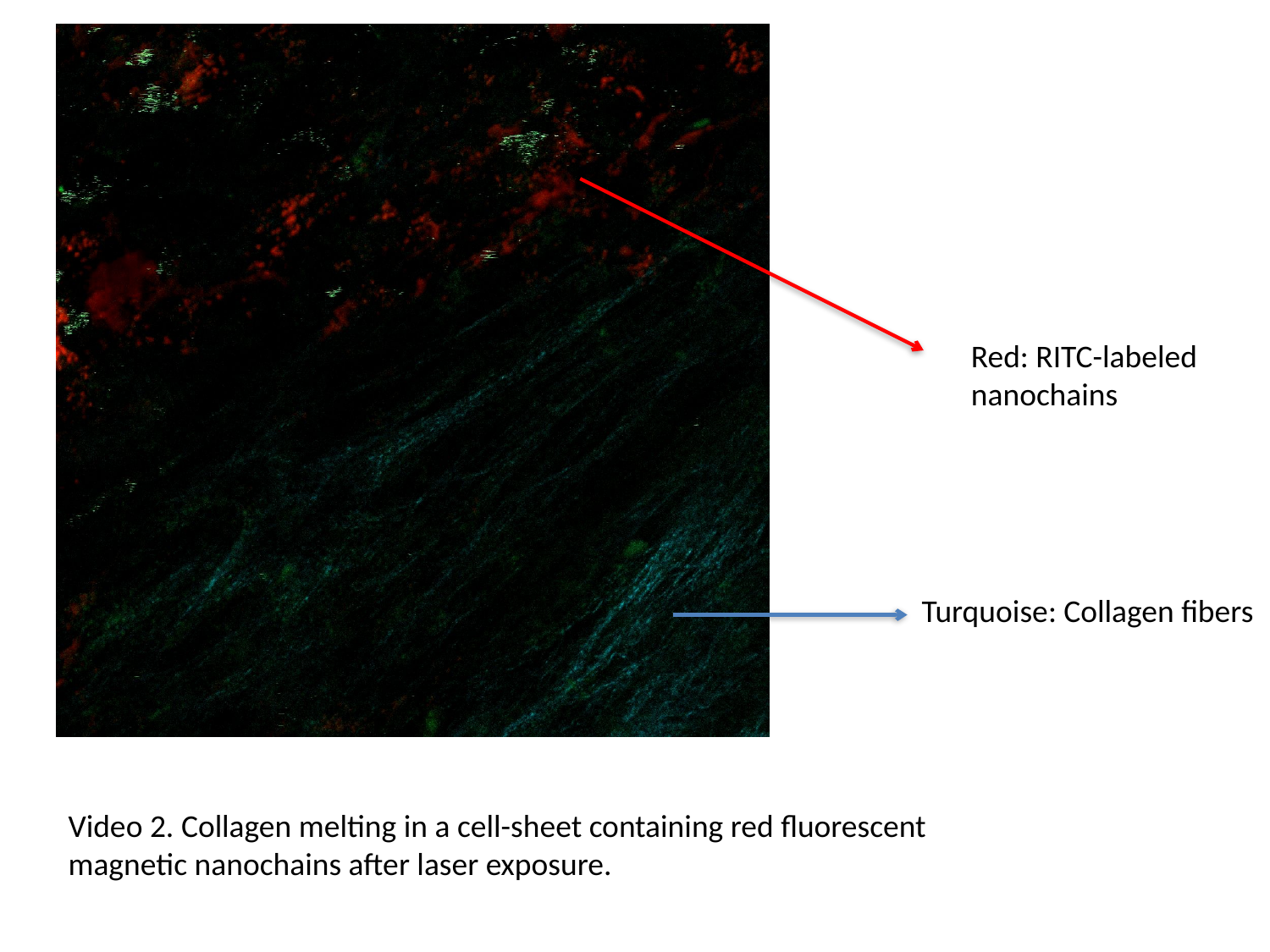

Red: RITC-labeled nanochains
Turquoise: Collagen fibers
Video 2. Collagen melting in a cell-sheet containing red fluorescent magnetic nanochains after laser exposure.
